# Gravitational Field effects on the Decoherence Process and the Quantum Speed Limit

**DOI:** 10.1038/s41598-017-15114-0

**Published:** 2017-11-08

**Authors:** Sh. Dehdashti, Z. Avazzadeh, Z. Xu, J. Q. Shen, B. Mirza, H. Wang

**Affiliations:** 1 Institute of Marine Electronics Engineering, Ocean College, Zhejiang University, Hangzhou, 310058 China; 2 The Innovative Institute of Electromagnetic Information and Electronic Integration, College of Information Science and Electronic Engineering, Zhejiang University, Hangzhou, 310027 China; 3 School of Mathematical Sciences, Nanjing Normal University, Nanjing, 210023 China; 4 Centre for Optical and Electromagnetic Research, State Key Laboratory of Modern Optical Instrumentations, Zijingang Campus, Zhejiang University, Hangzhou, 310058 China; 5 Department of Physics, Isfahan University of Technology, Isfahan, 84156-83111 Iran

## Abstract

In this paper we use spinor transformations under local Lorentz transformations to investigate the curvature effect on the quantum-to-classical transition, described in terms of the decoherence process and of the quantum speed limit. We find that gravitational fields (introduced adopting the Schwarzschild and anti-de Sitter geometries) affect both the decoherence process and the quantum speed limit of a quantum particle with spin-1/2. In addition, as a tangible example, we study the effect of the Earth’s gravitational field, characterized by the Rindler space-time, on the same particle. We find that the effect of the Earth’s gravitational field on the decoherence process and quantum speed limit is very small, except when the mean speed of the quantum particle is comparable to the speed of light.

## Introduction

Quantum mechanics imposes a limitation on the evolution of quantum systems. This limitation has two roots: one is a dynamical process, and the other environmental. The former is related to the fact that the time required for a given pure state to become orthogonal to itself under unitary dynamics is imposed as a boundary on the speed of evolution; such a boundary is intimately related to the concept of quantum speed limit (QSL), *τ*_*min*_^1^. Indeed, earlier studies have indicated that whenever the dynamics of the quantum system under study is governed by a Hermitian Hamiltonian, *H*, then the QSL *τ*_*min*_ has a lower boundary proportional to the inverse of the variance in the energy, ${\rm{\Delta }}H=\sqrt{\langle {H}^{2}\rangle \,-\,{\langle H\rangle }^{2}}$, i.e., *τ*_*min*_ ≥ *πħ*/2Δ*H* ^1^. The second reason for the existence of a QSL is related to the fact that quantum systems are ultimately coupled to their environment^2–4^. Therefore, applying QSL first requires a quantification of the effects associated with the environment. For this reason, an analogous boundary has been investigated in many open quantum systems^2–18^. Such a boundary on the evolution of an open system would help to address the robustness of the quantum systems that are applied, for example, against decoherence in simulators and in quantum computers^19^. Currently, the applications of these limits cover remarkably different scenarios, including quantum communication, identification of precision bounds in quantum metrology, formulation of computational limits of physical systems, and development of quantum optimal control algorithms^20–24^.

On the other hand, the quantum-to-classical transition has been one of the most important subjects in the foundation of physics, mathematical physics and philosophy of physics, and it will have an important role in technological applications^25^. The origin of the quantum-to-classical transition is formulated via an environmental effect, the so-called decoherence phenomenon. The effect of environment in the decoherence phenomenon has been modeled by three scenarios. In the first scenario, as a result of the environmental interaction with a quantum system, the environment gets entangled with the quantum system itself; therefore, the interaction of the environment with the quantum system leads to the decoherence process^25–29^, one example of environmental effect is the gravitational effect; for details see refs^30–36^. In the second scenario, the decoherence phenomenon is the result of fluctuations of the environment^37,38^. In the third scenario, the gravitational fields, described in general relativity by the space-time curvature, are at the origin of the decoherence process^39–43^. The latter scenario has been considered to be potentially relevant on two levels: on one side, it is an effect of the environment; on the other, it is one of the results obtained by changing the dynamical process of the quantum system in a curved environment. Consequently, studying the effect of gravitational fields on quantum systems is a fundamental problem.

In this paper we use spinor transformations under local Lorentz transformations to study the effects of the gravitational fields and the mean velocity of quantum particles on the quantum-to-classical transition, i.e., the decoherence process, and we investigate the role of different space-time backgrounds in this phenomenon. Indeed, we consider a superposition of up- and down-spin system and study the dynamical process of the system, while it moves into the curved space-time background. We show that the gravitational field, intended as the space-time background, causes an extra effect on the quantum dynamics of the system, namely on the quantum speed limit (QSL), which is a result of both the dynamical process and the environment. It is worth mentioning that the QSL, as a quantum phenomenon, is imposed to the system dynamics by the dynamical process and therefore it is different from the gravitational time dilation mentioned in ref.^30^. In addition, in order to examine the effect of the Earth’s gravitational field, we consider the weak gravitational field as the background space-time of the spin system. We study the dynamical process of a spin system in a Rindler space-time, which describes a uniformly accelerating particle framework. According to the equivalence principle of the general relativity of Einstein, which expresses gravitational fields are equal with accelerating frame of reference, we will study the impacts of gravitational fields on the decoherence process as well as quantum speed limit (QSL). We find that effects of the weak gravitational field, such as Earth’s gravity, is too difficult to detect, except when the mean velocity of the quantum system is comparable with the speed of the light.

## Results

### Wigner Rotation for circular geodesic motion in Schwarzschild-AdS geometry

We consider the Schwarzschild-AdS metric and we study the dynamical process of a spin system with an initial state as a superposition of the up and down spins. The calculations indicate that the corresponding reduced density matrix of the final states in the local inertial frame for a circulate motion with *r* = *const*. in an equatorial plane is given by

1 $${\rho }_{f}=\frac{1}{2}(\begin{array}{cc}1+\langle \sin {\rm{\Theta }}\tau \rangle  & \langle \cos {\rm{\Theta }}\tau \rangle \\ \langle \cos {\rm{\Theta }}\tau \rangle  & 1-\langle \sin {\rm{\Theta }}\tau \rangle \end{array}),$$

where 〈 〉 is the average of the wave function with respect to the distribution function and ${\rm{\Theta }}=csinh\zeta {cosh}^{2}\zeta$ $(1-\frac{{p}^{3}}{{p}^{0}+{m}_{0}c}\,tanh\zeta )L(r)$ with $L(r)=\sqrt{B(r)}(\frac{{\partial }_{r}B(r)}{2B(r)-\frac{B(r)}{r}})$ in which the function *B*(*r*) is characterized by the Schwarzschild radius *r*_*s*_ and Ads radius *l*, namely, $B(r\mathrm{)=1}-\frac{{r}_{s}}{r}+\frac{{r}^{2}}{{l}^{2}}$; *p* ^*i*^ is a component of the four-momentum and $\zeta ={\tanh }^{-1}[\frac{r}{c\,\sqrt{B(r)}}{\partial }_{t}\phi ]$. Notice that 〈cos Θ *τ*〉 = 0 causes the pure density matrix to be reduced to a mixed density matrix, i.e. it causes a quantum-to-classical transition.

**Figure 1:**
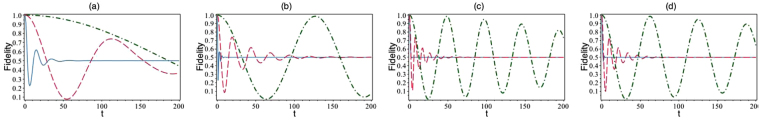
The fidelity ${\mathcal F}$ of the up and down superposition of a spin system as a function of time *t*, for different values of particle velocity, *v* = 0.1*c* (green dot-dashed line), *v* = 0.5*c* (red dashed line) and *v* = 0.9*c* (blue line). Plot (**a**) is set in the Schwarzschild background with *r*_*s*_ = 1 and *r* = 10.1. Plot (**b**) shows the same as (**a**), for *r*_*s*_ = 10 and *r* = 10.1. Plots (**c** and **d**) show the fidelity for AdS background with *l* = 1 and *r* = 1, and with *l* = 10 and *r* = 10 respectively; the color coding is the same as in plots (**a** and **b**). In the all Schwarzschild and AdS background plots, we choose *c* = 1 and *m* = 1.

**Figure 2:**
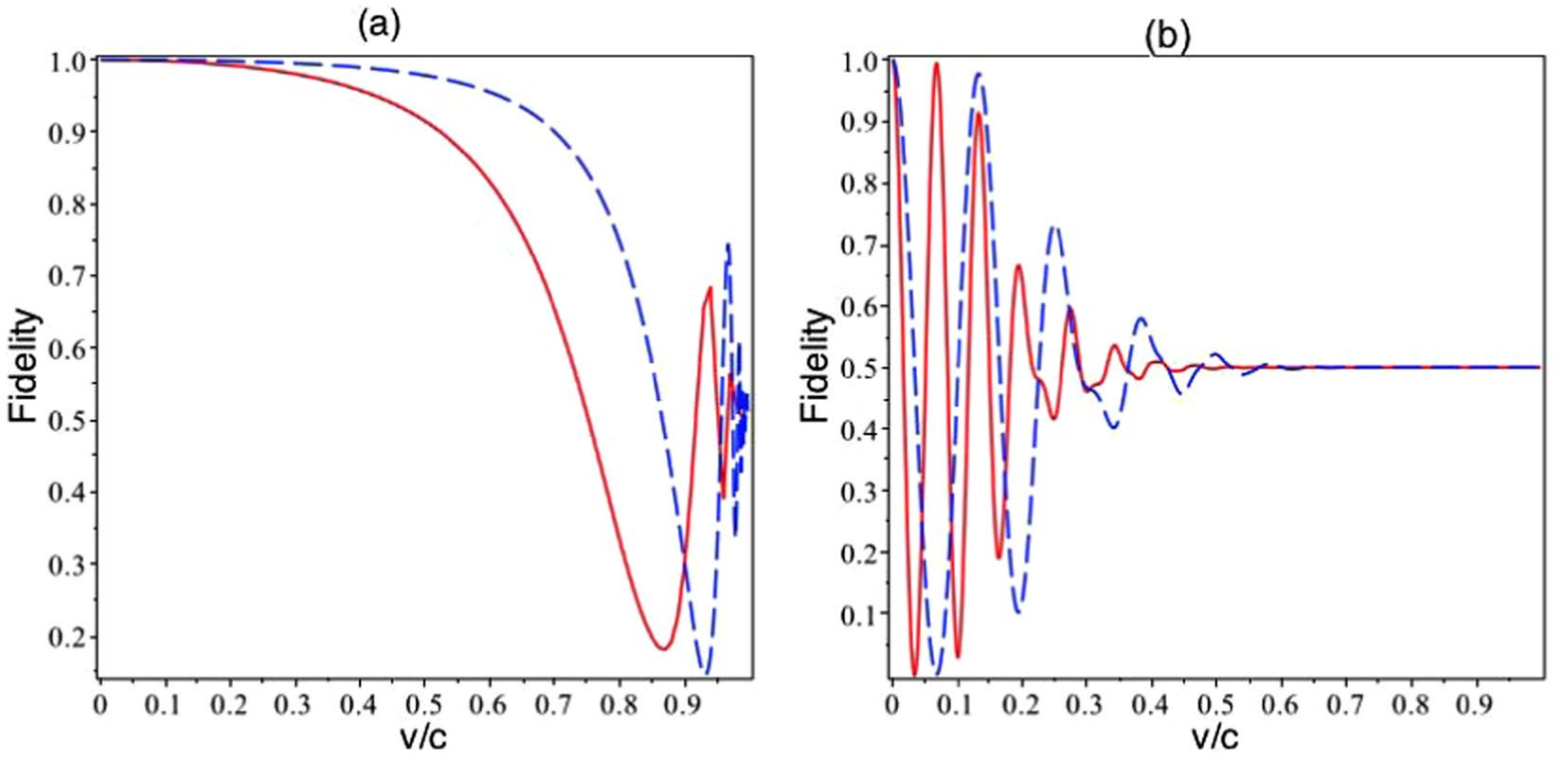
Plot (**a**) indicates the dependence of the fidelity ${\mathcal F}$ of the up and down spin superposition on the particle speed *v*/*c*, for *t* = *const*., i.e., *t* = 5 (dashed blue curve) and *t* = 10 (red curve), in the Schwarzschild space-time background with *r*_*s*_ = 10 and *r* = 10.1. Plot (**b**) shows the fidelity as a function of *v*/*c* for the same system, with the same parameters in the Ads background.

The fidelity ${\mathcal F}$, defined by ${\mathcal F} =Tr[{\rho }_{i}{\rho }_{f}]$, where *ρ*_*k*_, *k* = *i*, *f* are respectively the initial and final density matrices, is a convenient measure to know to what extent the evolution in time of the superposition state preserves coherence; according to definition, the fidelity ${\mathcal F}$ can be expressed as ${\mathcal F} =(1+\langle \cos \,{\rm{\Theta }}\tau \rangle )/2$, in which we will consider a Gaussian distribution for the average. Moreover, note that the off-diagonal elements of the density matrix is a function the fidelity, and we can consider the linear fidelity as a measure of the decoherence factor, i.e., $r(t)=\mathrm{|2} {\mathcal F} (t)\,-\,\mathrm{1|}$. Figure 1 shows the fidelity ${\mathcal F}$ (which, as is mentioned, can be considered as a measure of the decoherence factor) of an up- and down-spins superposition while it moves in a curved space-time. Plots (a) and (b) show the effects of the Schwarzschild background and of the mean velocity of the particle on the fidelity. These plots indicate that the fidelity has a behavior somewhat similar to that of a damped harmonic oscillator. Increasing the mean velocity of the particle causes the decoherence factor, i.e., 〈cos (Θ*τ*)〉, to approach rapidly zero. Figure 2a shows the fidelity as a function of the mean velocity. This figure confirms that increasing the mean velocity of the particle causes the fidelity to approach 1/2 in no time, when the mean velocity approaches the speed of light *c*; despite the fact that when the mean velocity of the particle almost touches the speed of light, we find fluctuation in its fidelity. Also, a comparison between plots 1-(a) and 1-(b) indicates that increasing *r*_*s*_, i.e. increasing the gravitational effect, causes the death and rebirth of the coherence of superposition as well as the damping behaviors of the fidelity in any specific time interval to be more pronounced. In brief, increasing the gravitational strength as well as the mean velocity of the quantum particle cause the decoherence factor, 〈*cos*Θ*τ*〉 to rapidly approach zero. In addition, plots 1-(c) and 1-(d) show the fidelity of the particle as it moves in the AdS background with different parameters. Also in these cases, increasing the mean velocity of the particle or increasing *l* cause the damping behavior and the rapid rise of the classical states to appear. Figure 2b indicates the impacts of variation of the mean velocity of the particle in the AdS-background. This figure also verifies the previous result, namely, increasing of the mean velocity of the particle causes the fidelity approaches 1/2; in the other word, the decoherence factor approaches zero in this situation and the role of geometry decreases, as a result of this fact, in both geometries, increasing mean velocity causes the decoherence factor to approach zero.

In addition, we study the QSL^4^, by using the fidelity. Indeed, by reparameterizing the fidelity, ${\mathcal F} =\,\cos \,\eta$, and considering |1 − cos*η*| ≤ 4*η* ^2^/*π* ^2^ ^4^, the QSL *τ*_*η*_ is given by:

2 $${\tau }_{\eta }\ge \frac{2{\eta }^{2}}{{\pi }^{2}\sqrt{|\langle {{\rm{\Theta }}}^{2}\rangle |}}$$

which is a function of the mean velocity of the quantum particle as well as of the properties of the geometrical background.

**Figure 3:**
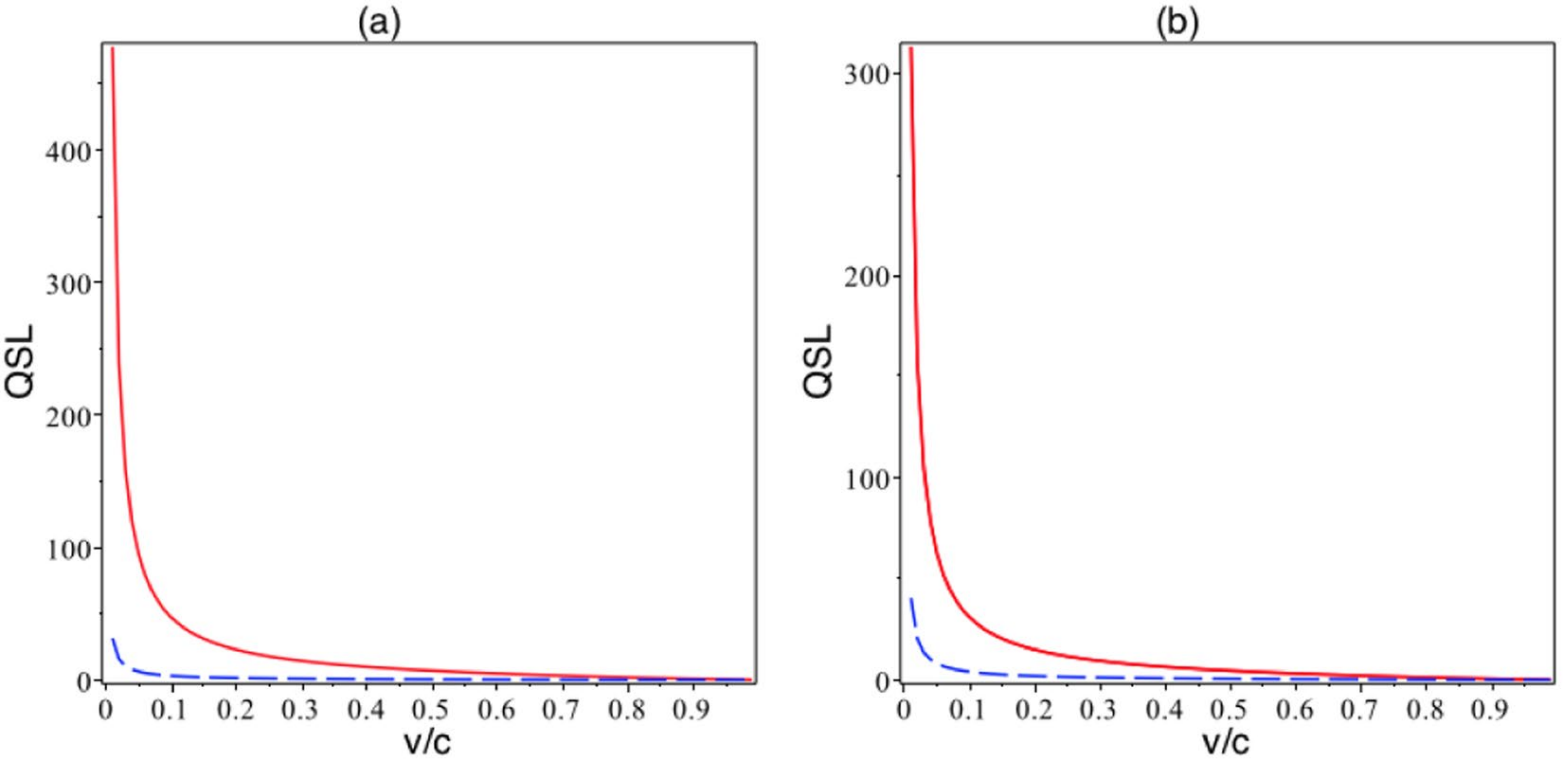
Variation of the QSL *τ*_*ζ*_ as a function of *v*/*c*, for different values of the radius *r*, *r* = 10 (red line), and *r* = 1.1 (blue dashed line) with *r*_*s*_ = 1 for the Schwarzchild space-time in plot (**a**); in plot (**b**) the variation of the QSL *τ*_*η*_ as a function of *v*/*c*, for different values of the radius *l*, *l* = 10 (blue dashed line), and *l* = 1 (red line), with *r* = 1, in both cases.

In Fig. 3, the variation of the QSL as function of the mean velocity *v*/*c* for a spin system in different gravitational field backgrounds are plotted. These plots indicate that increasing the mean velocity of the particle causes *τ*_*η*_ to decrease, which means that the speed of the dynamical process of the quantum system increases, in both cases, i.e., the Schwarzchild and AdS space-time. Moreover, in plots (a), one can see that the QSL *τ*_*η*_ increases by decreasing the gravitational field effect; in other words, the speed of the dynamical process of a quantum system is increased by the increasing the impacts of the background geometry and gravitational field. In the plot (b) variation of the QSL as a function of *v*/*c* in the AdS geometry is plotted. This plot illustrates that when the mean velocity of the quantum particle is comparable with the speed of light, the effects of geometry disappears, despite of the fact that when the mean velocity is not comparable with the speed of light, $v\ll c$, geometry is able to play role for control of QSL.

### Wigner Rotation for straight motion in Rindler space-time

In the previous sections we mathematically studied the effect of the gravitational field on the decoherence factor and on the QSL. In order to estimate the effect of the gravitational field in real life, we will consider the weak gravitational effect of the Earth, as a tangible example, by studying the Rindler space-time. We consider a quantum particle in motion along the *x*-axis, while the gravitational field is assumed to be along *z*-axis.

**Figure 4:**
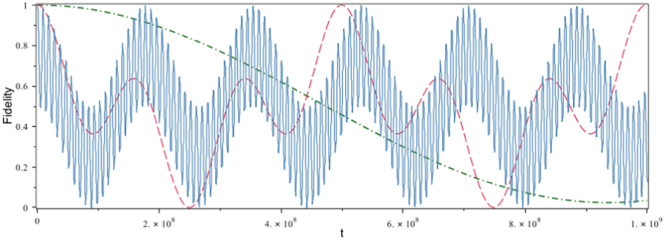
The fidelity as a function of *t*, for different values of velocity of an electron in the Rindler space-time background with *g* = 9.81(*m*/*s* ^2^).

**Figure 5:**
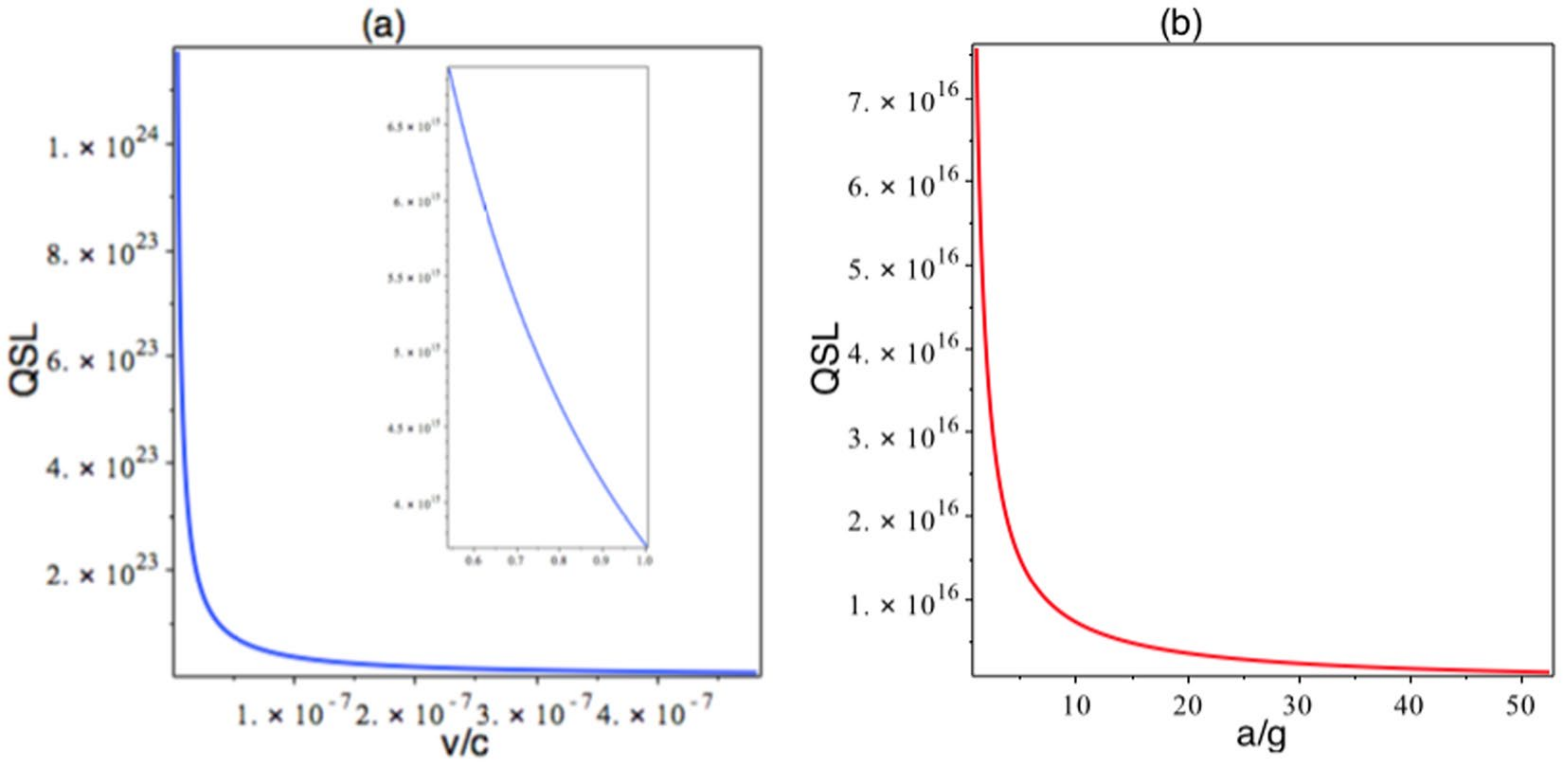
Variations of the QSL as a function of *v*/*c*, for the Rindler space-time with *g* = 9.81(*m*/*s* ^2^) in plot (**a**); Variations of the QSL as a function of *a*/*g*, for the Rindler space-time in plot (**b**).

By considering the initial state as a superposition of the up and down spins, one respectively obtains the fidelity ${\mathcal F}$ and the QSL *τ*_*η*_:

3 $$ {\mathcal F} =\frac{1}{2}+\frac{\langle {\cos {\rm{\Theta }}}_{R}\tau \rangle }{2},$$

and

4 $${\tau }_{\eta }\ge \frac{2{\eta }^{2}}{{\pi }^{2}\sqrt{|\langle {{\rm{\Theta }}}_{R}^{2}\rangle |}}\mathrm{.}$$

in which ${{\rm{\Theta }}}_{R}=-\frac{\frac{g}{c}}{1+\frac{gz}{{c}^{2}}}\,\sinh \,\zeta {\cosh }^{2}\zeta (1+\frac{{p}^{1}}{{p}^{0}+{m}_{0}c}\,\tanh \,\zeta )$, where *g* denotes the Earth’s acceleration and $\zeta ={\tanh }^{-1}\,[\frac{1}{c}(1+\frac{gz}{{c}^{2}})\frac{dx}{dt}]$. The effect of the Earth’s gravitational field on the fidelity for a spin system is shown in Fig. 4. For short times, the geometry almost does not affect the fidelity and the decoherence factor, except when the mean velocity of the particle is comparable with the speed of light. However, at large interval of time, the death and rebirth of superposition of the up and down spins are clearly evident. Figure 5 shows the variation of the QSL as functions of the mean velocity and acceleration of the particle in plots (a) and (b), respectively. Plot (a) in this figure indicates that the QSL is decreased by increasing the mean velocity of the particle, which means the speed of dynamical process of the system is increased. Also, by considering the relation (4), the QSL is proportional to the acceleration, ${\tau }_{\zeta }\propto \frac{c}{g}$, which means increasing the particle’s acceleration causes the QSL to decrease; in other words, the speed of dynamical process to increase, as the plot (b) points the fact out.

## Discussion

We studied the quantum decoherence process and the QSL by using spinors transformations under local Lorentz transformations. We found that for a quantum particle both the decoherence process and the QSL are generally affected by gravitational fields, as well as by the mean velocity of the quantum particle. We illustrated this fact by considering both properties of quantum system, i.e., quantum decoherence process and QSL, for the Schwarzschild and the AdS geometries. Moreover, we showed that in the Earth’s gravitational field modeled by the Rindler space-time the effect of the gravitational field is too weak, except when the mean velocity of the quantum particle is comparable with the speed of light. Moreover, we indicated that the evolution speed of the dynamical process is proportional to the acceleration of the quantum particle; therefore, the QSL is a quantum property that can be detected by local observers, at least when the mean velocity of the particle is comparable with the speed of light, in the convenient frameworks.

Although the present work focuses on study the gravitational effect on the quantum dynamics of a massive particle, it can also be extended to massless particles, such as photons. Since, compared with electrons, photon are less influenced by environmental noise, the effect of gravitationally-induced decoherence would be relatively easily detected for photons. Recently, by setting-up a coupled Stokes channel and an anti-Stokes one, twin photons, correlated with each other has been theoretically studied and experimentally verified^44^. This research indicates the correlation and squeezing between Stokes and anti-Stokes signals can be also switched by the relative nonlinear phase shift, which can play simulation role of curvature effects in this case. On the other aspect, one can study impacts of weak gravitational fields on the coupled equations of the creation and annihilation operators that give photon numbers of the Stokes and anti-Stokes field at the output site of the medium; indeed, this set-up could be considered as an experimental set-up for studying of influences of gravity on the decoherence process and QSL, if it is put into the suitable accelerator framework^44^.

## Methods

Given that the curved space-time background definition of a particle state is not unique^45^, we choose a local reference frame for observers so that the space-time is locally identical to the Minkowsky space-time. In this case, the states of the particle are locally well defined and the spinors transform under local Lorentz transformations^39,40^. Therefore, when a particle moves from a point *x* ^*μ*^ to a new point in the new local inertial frame *x* ^′*μ*^ = *x* ^*μ*^ + *u* ^*μ*^(*x*)*dτ*, the infinitesimal Wigner rotation is given by^39–41,43^, ${{W}^{a}}_{b}={\delta }_{b}^{a}+{{\vartheta }^{a}}_{b}d\tau ,$ where ${{\vartheta }^{0}}_{0}={{\vartheta }^{i}}_{0}={{\vartheta }^{0}}_{k}=0$ and

5 $${{\vartheta }^{i}}_{k}={{{\rm{\Lambda }}}^{i}}_{k}(x)+\frac{{{{\rm{\Lambda }}}^{i}}_{0}{p}_{k}-{\Lambda }_{k0}{p}^{i}}{{p}^{0}+{m}_{0}c},\quad i,k=1,2,3.$$

In the above mentioned relation the four-momentum *p* ^*i*^ in the local frame is defined by *p* ^*i*^(*x*) = *e* ^*i*^_*μ*_(*x*)[*mu* ^*μ*^(*x*)] for *i* = 0, 1, 2, 3; Λ_*b*_^*a*^ is given by

6 $${{{\rm{\Lambda }}}^{a}}_{b}=-\frac{1}{{m}_{0}{c}^{2}}[{a}^{a}(x){p}_{b}(x)\,-\,{p}^{a}(x){a}_{b}(x)]+{{{\boldsymbol{\chi }}}^{a}}_{b}(x),$$

where the four-acceleration in the local frame is *a* ^*a*^(*x*) = *e* ^*a*^_*μ*_(*x*)[*u* ^*ν*^(*x*)∇_*ν*_ *u* ^*μ*^(*x*)]; ***χ*** ^*a*^_*b*_(*x*) is defined by the curvature properties of the space-time background, i.e.,

7 $${{{\boldsymbol{\chi }}}^{a}}_{b}={u}^{\mu }(x)({{e}_{b}}^{v}(x){\nabla }_{\mu }{{e}^{a}}_{v}(x))\mathrm{.}$$

When the particle moves along a path *x* ^*μ*^(*x*), from *x*_*i*_^*μ*^ = *x* ^*μ*^(*τ*_*i*_) to *x*_*f*_^*μ*^ = *x* ^*μ*^(*τ*_*f*_), its wave function is given by |***ψ*** ^*f*^〉 = *U*(Λ(*x*))|***ψ*** ^*i*^〉, where the Lorentz transformation unitary operator *U*(Λ(*x*)) has a corresponding spin-1/2 irreducible representation ${D}_{mm\text{'}}^{\mathrm{1/2}}(W({\rm{\Lambda }}(x),p))$ ^46^.

### Wigner Rotation for circular geodesic motion in Schwarzschild-AdS geometry

We consider the Schwarzschild-AdS metric:

8 $$d{s}^{2}=-{c}^{2}B(r)d{t}^{2}+{B}^{-1}(r)d{r}^{2}+{r}^{2}(d{\theta }^{2}+{\sin }^{2}\theta d{\varphi }^{2}),$$

with $B(r)=1-\frac{{r}_{s}}{r}+\frac{{r}^{2}}{{l}^{2}}$, where *l* is the AdS radius and ${r}_{s}=\frac{2GM}{{c}^{2}}$ is the Schwarzschild radius. The tetrad fields are given by

9 $${{e}^{t}}_{0}=\frac{1}{c\sqrt{B(r)}},\,{{e}_{1}}^{r}=\sqrt{B(r)},\,{{e}_{2}}^{\theta }=\frac{1}{r},\,{{e}_{3}}^{\varphi }=\frac{1}{r\,\sin \,\theta }\mathrm{.}$$

We investigate the circular motion with *r* = const in the equatorial plane (*θ* = *π*/2), for which the tangent vector associated with this motion is

10 $${u}^{t}(x)=\frac{1}{\sqrt{B(r)}}\,\cosh \,\zeta ,\,{u}^{\varphi }(x)=\frac{c}{r}\,\sinh \,\zeta ,$$

where $\tanh \,\zeta =\frac{r}{c\sqrt{B(r)}}\frac{d\varphi }{dt}$. By substituting the tetrad fields defined in equation (9) into the definition of the acceleration, we find the only non-zero component of the acceleration, *a* ^1^:

11 $${a}^{1}=\frac{{c}^{2}{\cosh }^{2}\zeta }{\sqrt{B(r)}}[\frac{1}{2}{\partial }_{r}B(r)-\frac{B(r)}{r}{\tanh }^{2}\zeta ]\mathrm{.}$$

Moreover, from equation (7), the matrix *χ* ^*a*^_*b*_ can be written as:

12 $${{\chi }^{a}}_{b}=(\begin{array}{cccc}0 & {{\chi }^{1}}_{0} & 0 & 0\\ {{\chi }^{0}}_{1} & 0 & 0 & {{\chi }^{3}}_{1}\\ 0 & 0 & 0 & 0\\ 0 & {{\chi }^{1}}_{3} & 0 & 0\end{array}),$$

in which

13 $${{\chi }^{1}}_{0}={{\chi }^{0}}_{1}=-\frac{c}{2}\frac{{\partial }_{r}B(r)}{\sqrt{B(r)}}\,\cosh \,\zeta ,\quad {{\chi }^{3}}_{1}=-{{\chi }^{1}}_{3}=-\frac{c\sqrt{B(r)}}{r}\,\sinh \,\zeta \mathrm{.}$$

The unitary operator *U*(Λ^*a*^_*b*_(*x*)) corresponding to the local Lorentz transformation Λ^*a*^_*b*_(*x*) transforms the momentum eigenstate |*p* ^*a*^, *m*〉 into *U*(Λ^*a*^_*b*_(*x*))|*p* ^*a*^, *m*〉, i.e., *U*(Λ^*a*^_*b*_(*x*))|*p* ^*a*^, *m*〉 = ∑_*m*′_ *D*_*m*′*m*_(*W*(Λ(*x*), *p*)|Λ*p* ^*a*^, *m*′〉, in which *W*(Λ(*x*), *p*) is the Wigner rotation and *D*_*m*'*m*_(*W*(Λ(*x*), *p*) is its unitary representation. Finally, by using ${{\Lambda }^{0}}_{1}={\rm{c}}{\sinh }^{2}\zeta \,\cosh \,\zeta L(r)$ and ${{{\rm{\Lambda }}}^{1}}_{3}=-c\,\sin {\rm{h}}\zeta {\cos {\rm{h}}}^{2}\zeta L(r)$, in which

14 $$L(r)=\sqrt{B(r)}(\frac{{\partial }_{r}B(r)}{2B(r)}\,-\,\frac{B(r)}{r})\mathrm{.}$$

We obtain the Wigner equation, namely, *W*(Λ(*x*), *p*) = exp[−*iσ*_2_Θ*τ*], where *σ*_2_ is the component of the angular momentum operator along the *x* ^(2)^-axis of the local frame. Hence, the matrix *D* ^1/2^ can be represented as

15 $${D}^{1/2}(W({\rm{\Lambda }}(x)),p)=(\begin{array}{cc}\cos \,\frac{{\rm{\Theta }}\tau }{2} & \sin \,\frac{{\rm{\Theta }}\tau }{2}\\ -\sin \,\frac{{\rm{\Theta }}\tau }{2} & \cos \,\frac{{\rm{\Theta }}\tau }{2}\end{array}),$$

for which the circular geodesic motion Θ is obtained by

16 $${\rm{\Theta }}=c\,\sinh \,\zeta {\cosh }^{2}\,\zeta L(r)(1-\frac{{p}^{3}}{{p}^{0}+{m}_{0}c}\,\tanh \,\zeta )\mathrm{.}$$

Considering the initial state as a superposition of the up and down spins,

17 $$|{\psi }^{i}\rangle =\int {d}^{3}p\frac{{m}_{0}c}{\sqrt{|{\bf{p}}{|}^{2}+{m}_{0}^{2}{c}^{2}}}\frac{f({\bf{p}})}{\sqrt{2}}|{\bf{p}}\rangle \otimes (|+\rangle +|-\rangle ),$$

under the following condition

18 $$\int {d}^{3}p\frac{{m}_{0}c}{\sqrt{|{\bf{p}}{|}^{2}+{m}_{0}^{2}{c}^{2}}}|f({\bf{p}}{)|}^{2}=\mathrm{1,}$$

the corresponding final state in the local inertial frame is given by

19 $$|{\psi }^{f}\rangle =\int {d}^{3}p\frac{{m}_{0}c}{\sqrt{|{\bf{p}}{|}^{2}+{m}_{0}^{2}{c}^{2}}}\frac{f({\bf{p}})}{\sqrt{2}}|{\rm{\Lambda }}{\bf{p}}\rangle \otimes [(\cos \,\frac{{\rm{\Theta }}\tau }{2}-\,\sin \,\frac{{\rm{\Theta }}\tau }{2})|+\rangle +(\cos \,\frac{{\rm{\Theta }}\tau }{2}+\,\sin \,\frac{{\rm{\Theta }}\tau }{2})|-\rangle ]\mathrm{.}$$

The reduced density matrix of the final states is given by (1), where 〈 〉 is the average of the wave function with respect to the distribution function. Notice that 〈cos Θ*τ*〉 = 0 causes the pure density matrix (1) to be reduced to a mixed density matrix, i.e. it causes a quantum-to-classical transition.

### Effect of Geometry on Fidelity

The fidelity ${\mathcal F}$ is defined by ${\mathcal F} =Tr[{\rho }_{i}{\rho }_{f}]$, where *ρ*_*k*_, *k* = *i*, *f*, are respectively the initial and final density matrices. The fidelity is a convenient measure to know to what extent the evolution in time of the superposition state preserves coherence; in other words, the fidelity criterion is a measure of the decoherence factor. By using relation (1), the fidelity ${\mathcal F}$ can be expressed as

20 $$ {\mathcal F} =\frac{1}{2}+\frac{1}{2}\langle \cos \,{\rm{\Theta }}\tau \rangle \mathrm{.}$$

### QSL

To study the QSL, we consider the time derivative of the geometric Bures angle, ${\mathcal L} ({\rho }_{s}\mathrm{(0)},{\rho }_{s}(\tau ))$ $=\,\arccos (\sqrt{\langle \psi \mathrm{(0)|}{\rho }_{s}(\tau )|\psi \mathrm{(0)}\rangle })$, between the initial and final states of the quantum system^4^. The latter saturates the following inequality,

21 $$\frac{d}{dt} {\mathcal L} (\rho \mathrm{(0)},{\rho }_{s}(\tau ))\le |\frac{d}{dt} {\mathcal L} (\rho \mathrm{(0)},{\rho }_{s}(\tau ))|,$$

which leads to the following inequality^4^: $2\,\cos \, {\mathcal L} \,\sin \, {\mathcal L} \dot{ {\mathcal L} }\le ||\dot{\rho }(t)|{|}_{op}$, in which $||\dot{\rho }(t)|{|}_{op}$ denotes the operator norm of the operator $\dot{\rho }(t)$. Therefore, the QSL is given by

22 $$\tau \ge \frac{1\,-\, {\mathcal F} (\tau )}{{{\rm{\Lambda }}}_{op}},$$

in which $${{\rm{\Lambda }}}_{op}=\frac{1}{\tau }{\int }_{0}^{\tau }||\dot{\rho }(t)|{|}_{op}$$.

### Effect of Geometry on QSL in a Schwarzschild-AdS geometry

Now, By using the relation (1), we can obtain $||\dot{\rho }(t)|{|}_{op}=\sqrt{{\langle {\rm{\Theta }}\cos {\rm{\Theta }}\tau \rangle }^{2}+{\langle {\rm{\Theta }}\sin {\rm{\Theta }}\tau \rangle }^{2}}$. According to the following relation

23 $$||\dot{\rho }(t)||\le |\langle {\rm{\Theta }}\,\sin \,{\rm{\Theta }}\tau \rangle |+|\langle {\rm{\Theta }}\,\cos \,{\rm{\Theta }}\tau \rangle |,$$

and using the Cauchy-Schwarz inequality, we can obtain:

24 $${\rm{\Lambda }}=2\sqrt{|\langle {{\rm{\Theta }}}^{2}\rangle |}\ge {{\rm{\Lambda }}}_{op},$$

hence, by reparameterizing the fidelity, ${\mathcal F} =\,\cos \,\eta$, and considering |1 − cos *η*| ≤ 4*η* ^2^ */π* ^2^, the QSL _*τη*_ is given by the relation (2)^4^.

### Wigner Rotation for straight motion in Rindler space-time

In order to estimate the effect of the gravitational field in the level of Earth’s gravitational fields, we study the Rindler space-time:

25 $$d{s}^{2}=-{c}^{2}{(1+\frac{gz}{{c}^{2}})}^{2}d{t}^{2}+d{x}^{2}+d{y}^{2}+d{z}^{2}\mathrm{.}$$

In this case, the tetrads are given by ${{e}_{0}}^{t}={c}^{-1}{(1+\frac{gz}{{c}^{2}})}^{-1}$, ${{e}_{1}}^{x}=\mathrm{1,}$, ${{e}_{2}}^{y}=1$ and ${{e}_{3}}^{z}=1$. The tangent vectors associated with this motion are given by ${u}^{t}(x)=\,\cosh \,\zeta /(1+\frac{gz}{{c}^{2}})$ and *u* ^*x*^(*x*) = *c* sinh*ζ*. Hence, the only non-zero component of the four-acceleration is given by ${a}^{3}=\frac{g}{1+\frac{gz}{{c}^{2}}}{{\rm{c}}{\rm{o}}{\rm{s}}{\rm{h}}}^{2}\zeta$, while the only non-zero components of the tensor (7) are

26 $${\chi }_{3}^{0}={\chi }_{0}^{3}=-\frac{\frac{g}{c}}{1+\frac{gz}{{c}^{2}}}\,\cosh \,\zeta ,$$

and consequently, the non-zero components of the tensor (6) are given by

27 $${{\rm{\Lambda }}}_{3}^{0}=\frac{\frac{g}{c}}{1+\frac{gz}{{c}^{2}}}{\sinh }^{2}\zeta \,\cosh \,\zeta ,\quad {{\rm{\Lambda }}}_{3}^{1}=-\frac{\frac{g}{c}}{1+\frac{gz}{{c}^{2}}}\,\sinh \,\zeta {\cosh }^{2}\zeta \mathrm{.}$$

Finally, the Wigner equation is obtained *W*(Λ(*x*), *p*) = exp[−*iσ*_2_Θ_*R*_ *τ*], in which *σ*_2_ is the component of the angular momentum operator along the *x* ^2^-axis of the local frame. Therefore, we can write the matrix representation of *D* ^1/2^ as

28 $${D}^{1/2}(W({\rm{\Lambda }}(x)),p)=(\begin{array}{cc}\cos \,\frac{{{\rm{\Theta }}}_{R}\tau }{2} & \sin \,\frac{{{\rm{\Theta }}}_{R}\tau }{2}\\ -\sin \,\frac{{{\rm{\Theta }}}_{R}\tau }{2} & \cos \,\frac{{{\rm{\Theta }}}_{R}\tau }{2}\end{array}),$$

in which

29 $${{\rm{\Theta }}}_{R}=-\frac{\frac{g}{c}}{1+\frac{gz}{{c}^{2}}}\,\sinh \,\zeta {\cosh }^{2}\,\zeta (1+\frac{{p}^{1}}{{p}^{0}+{m}_{0}c}\,\tanh \,\zeta )\mathrm{.}$$

Finally, by considering the initial state as a superposition of the up and down spins, i.e., relation (17), we obtain the final state in the local inertial frame:

30 $$|{\psi }^{f}\rangle =\int {d}^{3}p\frac{{m}_{0}c}{\sqrt{|{\bf{p}}{|}^{2}+{m}_{0}^{2}{c}^{2}}}\frac{f({\bf{p}})}{\sqrt{2}}|{\rm{\Lambda }}{\bf{p}}\rangle \otimes [(\cos \,\frac{{{\rm{\Theta }}}_{R}\tau }{2}-\,\sin \,\frac{{{\rm{\Theta }}}_{R}\tau }{2})|+\rangle +(\cos \,\frac{{{\rm{\Theta }}}_{R}\tau }{2}+\,\sin \,\frac{{{\rm{\Theta }}}_{R}\tau }{2})|-\rangle ]\mathrm{.}$$

Following the same steps outlined in the previous sections, one obtains the fidelity ${\mathcal F}$ and the QSL _*τη*_ in case of the Rindler space-time as relations (3) and (4).
